# OCTA-derived retinal microvascular biomarkers in neuromyelitis optica spectrum disorder: clinical potential, methodological challenges, and possible links to astrocytopathy

**DOI:** 10.3389/fmed.2026.1880872

**Published:** 2026-07-31

**Authors:** Efstratia Amaxilati, Christos Bakirtzis, Angeliki Fountouki, Chrysoula Koutsiouki, Demetrios Pirounides

**Affiliations:** 1 Department of Ophthalmology, AHEPA University Hospital, Thessaloniki, Greece; 2 2nd Department of Neurology, AHEPA Hospital, Faculty of Health Sciences, Aristotle University of Thessaloniki, Thessaloniki, Greece; 3 Ophthalmica Eye Institute, Thessaloniki, Greece; 4 St George Hospital, Moorfields Eye Hospital, London, United Kingdom

**Keywords:** aquaporin-4, biomarkers, foveal avascular zone, MOGAD, multiple sclerosis, vessel density, neuromyelitis optica spectrum disorder, optical coherence tomography angiography

## Abstract

**Background:**

Neuromyelitis optica spectrum disorder (NMOSD) is an autoimmune demyelinating condition of the central nervous system that chiefly affects the optic nerves and spinal cord. Optical coherence tomography angiography (OCTA), a non-invasive imaging method for viewing the retinal microvasculature, offers potential biomarkers for early detection, monitoring disease activity, and distinguishing NMOSD from multiple sclerosis (MS) and myelin oligodendrocyte glycoprotein antibody-associated disease (MOGAD).

**Aim:**

To determine the potential of OCTA-derived retinal biomarkers in NMOSD and determine their clinical impact in diagnosis, disease monitoring, and therapeutic assessment.

**Methods:**

*A* narrative review of PubMed-indexed studies was conducted, including NMOSD patient’s diagnosed according to the 2015 international consensus criteria. Κey OCTA parameters—radial peripapillary capillary density, macular vessel density, perfusion density, and foveal avascular zone (FAΖ)—were analyzed across NMOSD subgroups with and without optic neuritis and compared to healthy controls.

**Results:**

NMOSD patients were found to have reduced vessel density in the superficial macular and radial peripapillary capillary plexuses regardless of their history of optic neuritis. These findings suggest possible subclinical retinal involvement. FAZ findings were inconsistent across studies, with reports of enlargement, reduction, or no significant difference. Reduced vessel density was also demonstrated to be correlated with poorer visual acuity, higher expanded disability status scale (EDSS) scores, and altered visual cortex connectivity.

**Conclusion:**

OCTA provides complementary information on retinal microvascular integrity in NMOSD and may help identify subclinical retinal involvement, particularly in the eyes without previous optic neuritis. Vessel density parameters, especially in the superficial macular and radial peripapillary capillary networks, appear more consistent than FAZ measurements, which remain heterogeneous and exploratory. OCTA may also contribute to multimodal differentiation among NMOSD, MS, and MOGAD, but it should not be considered a stand-alone diagnostic or monitoring tool. Standardized longitudinal studies with antibody stratification and rigorous confounder control are needed before OCTA-derived biomarkers can be incorporated into routine NMOSD decision-making.

## Introduction

Neuromyelitis optica (NMO), also known as Devic’s disease, is a severe autoimmune condition of the central nervous system (CNS) with prominent involvement of the spinal cord (1–3). It was first described in 1894 as the simultaneous attack of optic neuritis and transverse myelitis (3). These patients fall into the category of monophasic NMO, in which only one episode occurs over a period of more than 5 years and accounts for about 10% of cases (1, 4).

On the other hand, the main pattern of NMO is characterized by recurrent episodes, even years apart, of transverse myelitis and optic neuritis, involving nearly 90% of patients (1, 4). Εach attack leaves NMO patients with residual damage that gradually leads to disability after multiple episodes, such as blindness. This course of the disease contrasts with other demyelinating disorders, like multiple sclerosis (MS), where disability typically appears in the later stages (1, 5).

Apart from specific clinical findings of NMO, laboratory tests along with spinal cord imaging facilitate the differential diagnosis of the disease (2). In more detail, neuroimaging reveals lesions of transverse myelitis in more than three consecutive vertebral segments, whereas brain lesions are usually absent during the early course of the disease or are non-specific and clinically silent (1, 6, 7). Additionally, autoantibodies of aquaporin-4 (AQP4-IgG) are commonly detected in the blood samples of NMO patients, making this test highly indicative of the disease (8, 9). The percentage of seropositive cases in NMO is between 75 and 90% (2). This observation, made in 2004, led to the revision of the diagnostic criteria for NMO in 2006 and marked a breakthrough in the understanding of the condition (8–10).

Thus, in contrast to the initial impression, since 2007, NMO has been recognized as a broader spectrum of clinical manifestations and is known as neuromyelitis optica spectrum disorder (NMOSD) (2). More precisely, NMOSD encompasses not only typical NMO cases but also those with distinct brain lesions, NMO-seropositive patients with autoimmune disorders, such as systemic lupus erythematosus, NMO-seropositive patients with atypical NMO symptoms, and patients with optico-spinal MS (2, 11). NMOSD is primarily associated with AQP4-IgG (8, 9, 11). Additionally in 2015, the diagnostic criteria for NMOSD were formally refined through an international consensus, dividing patients into seropositive and seronegative NMOSD groups, with stricter diagnostic criteria for the latter group requiring clinical presentations associated with specific neuroimaging lesions. Moreover, while seropositive patients require only one of the six main regions of the CNS to be affected for the diagnosis, seronegative patients need two, with at least one located in the optic nerve, spinal cord, or the dorsal medullary area postrema (11). Εarly and accurate diagnosis is critical for effective management and prognosis (11).

In addition to aquaporin-4 seropositive (AQP4+) and seronegative (AQP4-) NMOSD, myelin oligodendrocyte glycoprotein antibody-associated disease (MOGAD) is now recognized as a distinct antibody-mediated inflammatory disorder of the CNS (11, 12). Since MOGAD may overlap clinically with NMOSD and MS, particularly through optic neuritis and myelitis, differentiating AQP4 + NMOSD, MOGAD, double-seronegative disease, and MS is an important clinical priority (12). Optical coherence tomography angiography (OCTA) may contribute to this multimodal differentiation by characterizing retinal microvascular patterns when interpreted together with clinical history, magnetic resonance imaging (MRI), antibody testing, visual function, and structural optical coherence tomography (OCT) (13).

All these at-risk regions of the CNS exhibit similar histopathological changes in NMOSD patients, including inflammation, perivascular infiltration, and axonal loss (14). Thus, the retina could serve as an accessible window for assessing central nervous system microvascular and neuroaxonal integrity (15, 16). OCT and OCTA are two non-invasive and rapid methods of visualizing the state of neurons and blood vessels, respectively, in the retina with potential applicability to the assessment of broader neuroinflammatory disease processes (16). Recent studies suggest that NMOSD affects both the superficial and deep retinal vascular plexuses, leading to measurable perfusion deficits (17). Aquaporin-4 (AQP4) is expressed on Müller cells and astrocytic endfeet surrounding retinal vessels (18–20). Therefore, OCTA may visualize microvascular changes related to astrocytic injury in NMOSD (18, 20, 21). Additionally, OCTA could play a role in differentiating NMOSD from MS, as distinct vascular patterns have been reported in each disorder (22, 23).

This review aims to offer a thorough critical analysis of the existing literature on OCTA-derived retinal biomarkers in patients with NMOSD. In addition to summarizing reported vascular findings, it evaluates whether OCTA abnormalities represent secondary changes following optic neuritis (ON) or reflect a primary manifestation of NMOSD-related astrocytopathy. To address this question, the review compares study design, OCTA platform and scan protocol, segmentation strategy, definitions of optic neuritis, AQP4 serostatus and outcome measures across the included studies. This approach allows the evidence to be interpreted in relation to the central mechanistic question of whether retinal microvascular rarefaction reflects AQP4-IgG-related astrocytic/Muller cell injury, secondary damage after optic neuritis, or methodological heterogeneity (18, 24). By integrating methodological differences with mechanistic interpretation, this review clarifies the current clinical value and limitations of OCTA as a potential diagnostic and monitoring biomarker in NMOSD. Comparative OCTA findings in MS and MOGAD are also considered to determine whether the reported microvascular alterations are specific to NMOSD or shared across inflammatory demyelinating disorders.

## Literature search and study selection

This narrative review was based on a structured search of PubMed/MEDLINE for studies evaluating OCTA-derived retinal or optic-disc microvascular parameters in NMOSD, with additional consideration of comparative data from MS and MOGAD. The search included studies published up to 2026 using combinations of the terms “neuromyelitis optica spectrum disorder,” “NMOSD,” “aquaporin-4,” “AQP4,” “optical coherence tomography angiography,” “OCTA,” “vessel density,” “radial peripapillary capillary vessel density,” “macular vessel density,” “foveal avascular zone,” “multiple sclerosis,” “MS,” “myelin oligodendrocyte glycoprotein,” and “MOGAD.” Eligible studies included patients with NMOSD diagnosed according to accepted diagnostic criteria and reported OCTA-derived vascular parameters. Comparative studies involving MS or MOGAD were also considered when relevant to differential diagnosis. Articles focused exclusively on structural OCT without OCTA, and non-human studies, case reports, and non-English articles were excluded. As this was a narrative review, no quantitative synthesis or formal risk-of-bias assessment was performed.

## Characteristics of included studies

**Figure 1:**
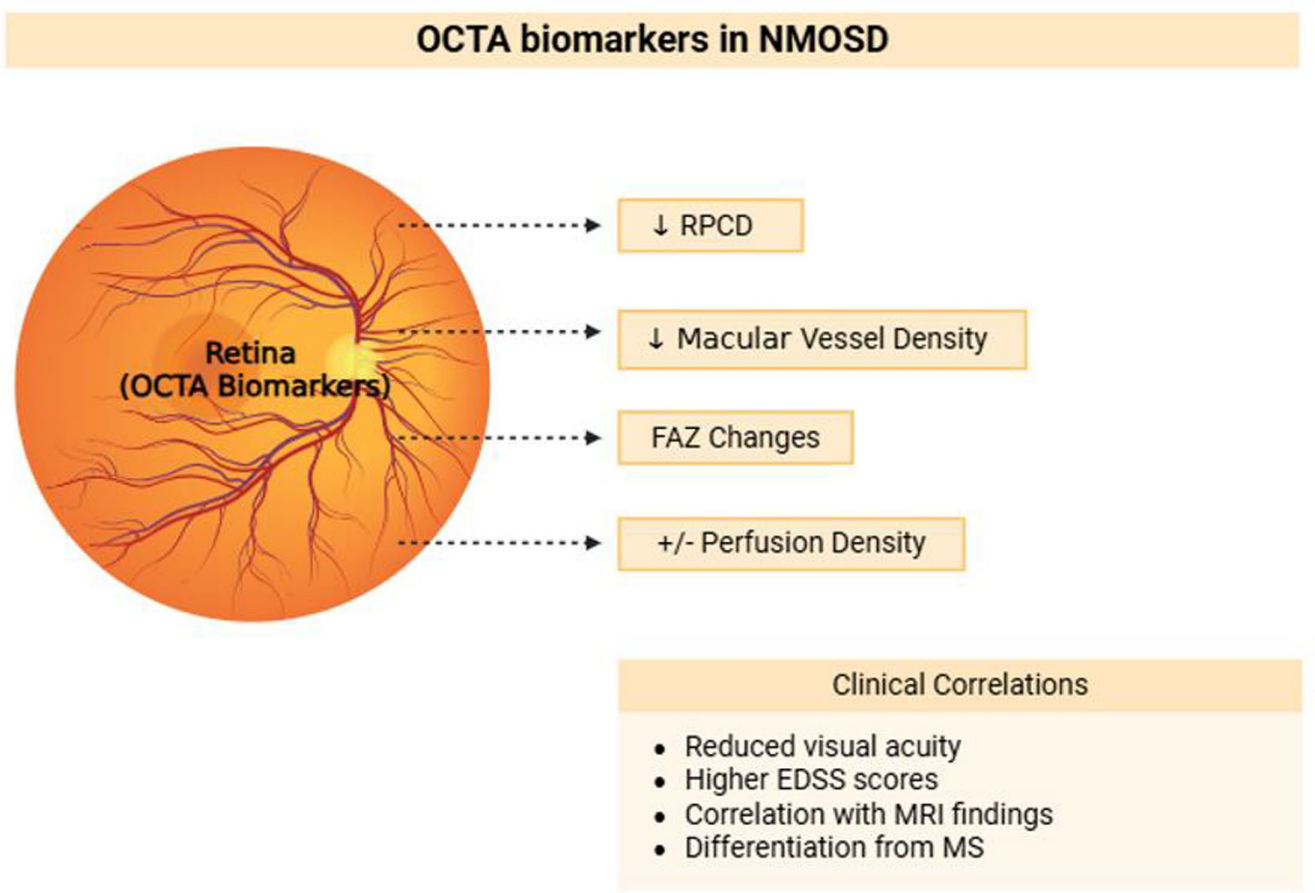
Overview of OCTA biomarkers in NMOSD and their associations with visual acuity, EDSS score, MRI findings, and their potential use in differentiating NMOSD from multiple sclerosis (MS).

In this review, most identified studies were cross-sectional, except Liu et al. (25), which included longitudinal follow-up. All involved patients with NMOSD were diagnosed according to the revised 2015 criteria (11). In several studies, NMOSD patients were categorized into two groups based on their history of previous optic neuritis attacks and were compared with healthy controls. The demographic characteristics, such as age, were generally comparable across all study groups. The analyzed studies excluded patients with recent ON, commonly using a 6-month washout period before OCTA imaging. However, this criterion does not provide the actual time elapsed since the last ON attack, which was not reported in any of the analyzed studies. In addition, the number or frequence of previous ON attacks was reported inconsistently across studies. As OCTA parameters may be influenced by time from last ON, recurrent ON attacks, and disease duration, these factors should be considered when interpreting the findings. Among these variables, only the number or frequency of previous ON attacks could be extracted from the included studies and was therefore added to Table 1 where available. When these data were not reported, they were marked as “NR.” Some studies specifically included only AQP4 + patients (13, 17, 26–33). Most included studies used AngioVue, while a few used Spectralis and AngioPlex, which may account for minor discrepancies in the results (17, 21, 26–38). The key OCTA biomarkers in NMOSD identified in these studies are discussed below. Correlations between these biomarkers and clinical measures, such as visual acuity (VA), expanded disability status scale (EDSS) scores, and MRI findings, underscore their clinical utility. OCTA-derived vascular parameters may also support relapse risk assessment and evaluation of treatment response, although their role remains unvalidated. The principal OCTA-derived biomarkers and their clinical correlations are summarized in Figure 1.

**Table 1:** Summary of the key findings of the studies.

Study	NMOSD Patients (n)	ON+ Eyes (n)	ON- Εyes (n)	Number of ON attacks/ frequency	AQP4-IgG	MOG-IgG+	OCTA device/ platform	Scan protocol/region	Segmentation / vascular layer	Image-quality/artifact criteria	Excluded ocular/systemic confounders	OCTA Βiomarkers	Κey Results in NMOSD eyes
Aly et al. (21)	16	9	21	NR	10/16 AQP4+ 6/16 double seronegative	excluded	RTVue XR Avanti / Optovue OCTA	6 × 6 mm foveal OCTA scan	SCP/DCP density from 1–3 mm eccentricity; FAZ area	OCTA SSI ≥ 60; excluded incorrect segmentation, decentration, major motion artifacts; 2 NMOSD eyes excluded.	Eye disease affecting retinal architecture/ vasculature, prior eye surgery and refractive error >6 D	SCP/DCP VD, FAΖ	Reduced VD, larger FAΖ correlated with GFAP, ΕDSS and VA
Wei et al. (17)	34	42	26	Patient level None 13; Once 10; ≥2 episodes 11	Positive	NR	Cirrus 5,000 OCTA, Zeiss Meditec, version 10.0	3 × 3 mm macular scan; 2.5-mm diameter annulus around fovea excluding FAZ	SCP, DCP and WCP; U-net segmentation and fractal dimension/box-counting analysis	Signal quality ≥6; 12/46 NMOSD patients excluded due to poor image quality	Systemic or neurological disease affecting brain and retina	Fractal dimension SCP/DCP VD	Reduced SCP and DCP VD; correlates with ΕDSS and VA
Guo et al. (34)	22	25	19	NR	Positive	NR	RTVue XR Avanti OCTA, Optovue	3 × 3 mm foveal OCTA images; SVC, DVC and IVC en face images	Deep-learning/active-contour vessel and FAZ segmentation; 12 microvascular/FAZ metrics extracted	Low SSI, artifacts and obvious image problems excluded; automated segmentation framework used	Hypertension, neurological disorders and substance abuse	SCP/ICP/DCP VD, FAΖ shape, size	Reduced VD in ON + eyes; enlarged FAΖ; reduced DCP VD in ON- eyes
Rogaczewska et al. (26)	13	9	11	NR	Positive	NR	RTVue XR Avanti AngioVue v2017.1.0.151	4.5 × 4.5 mm optic nerve head scan; 1.0-mm annulus around disc; 8 sectors	RPC layer from ILM to outer RNFL boundary; automatic AngioVue segmentation	SSI ≥ 50; significant motion artifacts excluded	Myopia >6 D, optic disc drusen, hypertensive /diabetic retinopathy, glaucoma, uveitis, eye surgery	RPCD.	Reduced RPCD; more pronounced in ON+ eyes; helped distinguish NMOSD from MS.
Rogaczewska et al. (28)	13	9	11	NR	Positive	NR	RTVue XR Avanti AngioVue v2017.1.0.151	3 × 3 mm macular scan centered on foveola	Automatic SCP and DCP segmentation; device VD output; DCP–SCP discrepancy.	SSI ≥ 50; significant motion artifacts excluded.	Myopia >6 D, macular disease, hypertensive/ diabetic retinopathy, glaucoma, uveitis, eye surgery	DCP/SCP VD	Reduced SCP VD especially in ON+ eyes; DCP VD was relatively preserved
Kwapong et al. (37)	20	14	6	Eye level once 8 eyes; ≥2 episodes 6 eyes	NR	NR	RTVue XR Avanti AngioVue v2015.1.0.90	3 × 3 mm macular scan centered on fovea; 304 × 304 pixels; 2.5-mm annular analysis excluding 0.6-mm FAZ	SCP, DCP and WCP automatically segmented; custom vessel segmentation/thresholding; Bennett formula for axial length correction.	SSI > 40; motion correction; excluded double-vessel pattern or motion artifacts >3 lines.	Glaucoma, cataract, high myopia, amblyopia and other ophthalmologic disease; diabetes, MS and other systemic/neurologic disease; prior ocular surgery	SCP VD, DCP VD	Reduced SCP/DCP VD; correlated with lower VA
Lang et al. (13)	21	18	24	Median ON episodes 1.0 (range 0–5)	Positive	MoG-IgG analyzed separately	VG200S OCTA, SVision Imaging, version 2.1.016	3 × 3 mm macular OCTA area around fovea; 18 radial B-scans for structure	Automatic segmentation; SCP, ICP and DCP; FAZ metrics.	Image-quality criteria not extensively detailed in text; automatic segmentation by device	Confounding neurologic/ ophthalmic disorders, ocular surgery/trauma, refractive error ±6 D, uncontrolled hypertension/ diabetes, glaucoma	SCP/ICP/DCP VD, FAΖ	Reduced SCP/ICP/DCP VD (p < 0.05), enlarged FAΖ
Chen et al. (31)	27	21	26	Eye level 1 episode 9 eyes; ≥2 episodes 12 eyes	Positive	NR	RTVue-XR Avanti OCTA, Optovue; OCTExplorer for structural segmentation.	ONH 4.5 × 4.5 mm; macular 3-mm diameter annular zone	RPC from ILM to posterior RNFL; superficial macular layer from 3 μm below ILM to outer IPL boundary	Scan quality ≥6; residual motion artifacts excluded	IOP > 21 mmHg, high refractive error, ocular surgery or other eye disease (glaucoma or AMD); uncontrolled hypertension	SCP VD, RPCD	Reduced SCP VD and RPCD
Lee et al. (36)	37	47	0	NR	33/37 AQP4+ 4/37 double seronegative	excluded	Topcon DRI OCT Triton Plus swept-source OCTA; IMAGEnet 6 v1.14.8538	4.5 × 4.5 mm peripapillary and 3.0 × 3.0 mm perifoveal scans.	SCP 3 μm below ILM to 15 μm below IPL; DCP 15–70 μm below IPL; RPCD from ILM to posterior RNFL.	TopQ ≥60; excluded poor centration, >10% motion artifacts, poor visibility; disc/FAZ margin manually adjusted when needed; 5 eyes excluded.	Ocular pathology affecting visual function, retinal disease, glaucoma, compressive optic neuropathy, systemic vasculitis, malignancy and neurologic disease before first ON	SCP/DCP VD, RPCD	Reduced SCP, DCP and RPCD in ON+ eyes
Wang et al. (33)	19	NR	NR	Patient-level ON frequency 2.32 ± 1.89	Positive	excluded	Avanti RTVue-XR OCTA, Optovue, software v2017.100.0.1	ONH/peripapillary RPC imaging with 0.7-mm wide oval annulus	Automatic device segmentation and measurement of RPCD	Signal quality ≥6; details on artifact exclusion limited	Eye/systemic disorders causing optic fundus damage, retinal vascular damage, eye inflammation, structural CNS abnormalities, drug/alcohol addiction and MRI contraindications.	RPCD	Reduced RPCD; linked with lower visual cortex functional connectivity
Ma et al. (32)	19	19	0	Patient-level: none 2; once 5; ≥2 episodes 12	Positive	NR	Avanti RTVue-XR OCTA, Optovue, software v2017.100.0.1	Optic nerve head/peripapillary RPC imaging.	Device automatic segmentation and measurement of RPCD	Signal quality ≥6; OSCAR-IB criteria.	Disc swelling/ microaneurysm; Possible eye disease	RPCD	Lower RPCD; correlated with altered primary visual cortex connectivity
Zhang et al. (38)	124	113	95	NR	Positive	NR	Cirrus 5,000 / AngioPlex v10.0, Zeiss Meditec	6 × 6 mm macular OCTA; optic disc cube 200 × 200; macular cube 512 × 128	Superficial capillary plexus from ILM to IPL; VD/PD in 9 sectors after FAZ exclusion; FAZ metrics automatic.	FastTrac tracking; excluded signal strength <7, poor centration, segmentation errors and motion artifacts.	Other systemic diseases, high myopia/hyperopia (SE < −6 or > + 3 D), significant media opacity, age <18, previous ocular surgery and POAG in NMOSD.	Macular VD, PD, FAΖ	Lower macular VD and PD; smaller FAZ
Liu et al. (27)	91	90	45	NR	Positive	excluded	Cirrus 5,000 / AngioPlex v10.0, Zeiss Meditec	6 × 6 mm macular OCTA; pRNFL 3.46-mm disc circle; macular cube 512 × 128 for GCIPL	SCP; annular VD/PD sectors after FAZ exclusion.	Scan quality ≥7; residual motion artifacts excluded	Systemic diseases including hypertension /diabetes; glaucoma, cataract, myopia <−6 D, hyperopia >6 D and eye surgery.	VD, PD, FAΖ	Significant in differentiating NMOSD from MS
Tiftikcioglu et al. (40)	9	15	3	Patient level: Monophasic ON 2/9; recurrent ON 7/9	AQP4 + 44.4%	NR	Zeiss Cirrus HD-OCT 5000/500 with AngioPlex	6 × 6 mm OCTA scans of macular and peripapillary regions	SCP from ILM to IPL; superficial peripapillary and macular VD; FAZ area, perimeter and circularity.	Scans inspected by two independent clinicians; excluded segmentation errors, large floaters, blinking and motion artifacts. Numeric signal-quality threshold NR.	Vascular comorbidities; glaucoma, amblyopia, AMD, high refractive error, prior ocular surgery and cataract	Macular VD, RPCD, FAΖ	Reduced VD and RPCD, smaller FAΖ
Huang et al. (30)	55	52	56	NR	Positive	NR	Avanti RTVue-XR, Optovue, software v2017.100.0.1	Peripapillary 0.7-mm wide annulus outside disc boundary; parafoveal annular zone 0.6–2.5 mm diameter.	RPCD: ILM to NFL. SCP layer: 3 μm below ILM to outer IPL boundary; software-generated ETDRS sectors.	OCTA SSI ≥ 40; poor-quality or residual-motion images rejected; APOSTEL-aligned reporting.	Media opacity, pathological myopia, RVO, diabetic/ hypertensive retinopathy, AMD, glaucoma, NAION, congenital/fundus disease, ocular trauma/surgery, chronic systemic diseases, recent steroids, antihypertensive/antiarrhythmic drugs and toxic retinal drugs.	SCP VD, RPCD	Reduced SCP VD and RPCD in ON + and ON- eyes, correlated with worse visual function
Li et al. (35)	51	NR	NR	NR	Positive	NR	RTVue-XR AngioVue, Optovue	4.5-mm circle/4.5 × 4.5 mm optic disc scan; high-density Angio Retina 6.0-mm macular scan	RPCD automatically calculated; sectors around ONH	Same experienced technician; Motion Correction Technology and 3D projection artifact reduction; detailed SSI threshold NR.	Retinal or ocular conditions interfering with OCTA, prior refractive surgery, prior laser treatment, ocular surgery within 3 months	RPCD	Reduced RPCD; correlated with poor VA
Wang et al. (39)	62	44	77	NR	Positive	NR	AngioVue RTVue XR Avanti, Optovue	HD 6 × 6 mm macular/foveal angio-retina scan; HD 4.5 × 4.5 mm optic-disc scan; GCC and optic nerve head modes.	SCP and DCP vessel density automatically generated; SCP from ILM to near IPL/INL junction; DCP from near IPL/INL junction to OPL/ONL junction; optic-disc sectors generated by software.	Only scans with quality index ≥7/10 included.	Systemic diseases affecting CNS/retina, inability to complete examination, high myopia, macular degeneration, retinal detachment, vascular occlusion, glaucoma, uveitis, amblyopia, ocular surgery/trauma	SCP/DCP VD, RPCD	Reduced SCP/DCP VD and RPCD independent of ON; correlated with lower EDSS and VA
Dong et al. (42)	44	87	0	ON recurrence frequency reported by subgroup: 1 (1–4) and 1 (1–3)	AQP4 + and double seronegative	excluded	Cirrus HD-OCT 5000 Review Software V.10 / AngioPlex, Carl Zeiss Meditec	Angiography 6 × 6 mm macular and optic disc scans.	Superficial layer from ILM to IPL; optic disc and macular central/inner/outer zones. Deep retinal VD/PD not available due to software limitations.	OCTA scan image quality >4 required; examinations performed by experienced OCT technician.	Glaucoma, macular degeneration, severe visual impairment, severe lenticular opacity with refractive power > − 3.0 DS	Macular VD/PD, optic disc VD/PD, FAZ	AQP4 + NMOSD showed significantly reduced macular and optic-disc PD and VD
Liu et al. (25)	45	54	31	NR	Positive	NR	Avanti RTVue-XR / Optovue, software v2017.100.0.1; split-spectrum amplitude decorrelation algorithm	Peripapillary RPC 0.7-mm elliptical annulus from disc boundary; macular parafoveal annulus 0.6–2.5 mm around fovea.	RPCD between ILM and RNFL; macular SCP/DCP segmented as inner two-thirds and outer one-third of GCL/IPL; FAZ automatically determined.	OSCAR-MP and APOSTEL quality criteria; images reviewed by a blinded ophthalmologist; poor-quality OCTA excluded.	Severe glaucoma, AMD, confounding neurological disorders; severe cataract and macular edema; Cardiovascular/metabolic risk factors not formally controlled.	SCP/DCP VD, RPCD, FAZ	Reduced densities and enlarged FAZ at baseline. FAZ remained the same at follow-up; densities showed further reduction.

VD, Vessel density; SCP, Superficial capillary plexus; DCP, Deep capillary plexus; ICP, Intermediate capillary plexus; FAΖ, Foveal avascular zone; RPCD, Radial peripapillary capillary density; PD, Perfusion density.

## Radial peripapillary capillary density

Radial peripapillary capillary vessel density (RPCD) is one of the most extensively examined OCTA factors in NMOSD. It depicts the superficial vasculature, spreading around the optic disc. All studies evaluating the RPCD reported lower RPCD in NMOSD patients compared with healthy controls (26, 30–32, 35, 36, 39, 40). Moreover, many studies specifically investigated the difference in RPCD between NMOSD patients with and without previous episode of optic neuritis (ON+ and ON-, respectively), comparing them to healthy controls (25, 26, 30, 31, 35). Interestingly, eyes without a history of optic neuritis (ON-) demonstrated relatively preserved vessel density compared to those with previous optic neuritis attacks. However, NMOSD ON- eyes exhibited reduced vessel density when compared to healthy controls, indicating that retinal changes may occur even in subclinical phases of the disease (26, 30, 31). For example, Chen et al. (31) reported that retinal peripapillary capillary vessel density was 49% in NMOSD ON- eyes compared to nearly 38% in ON + eyes and 50.5% in healthy controls, (*p* < 0.001 and *p* < 0.029, respectively). On the contrary, similar studies by Li et al. (29) and Li et al. (35) did not reveal a statistically significant difference in RPCD between NMOSD ON- patients and healthy controls.

## Macular vessel density

Parafoveal vessel density was evaluated in its entirety and within the three distinct capillary plexuses: superficial (SCP), intermediate (ICP), and deep capillary plexuses (DCP). A lower capillary density of all three plexuses was reported in NMOSD patients compared to healthy controls in several studies (13, 17, 25, 27, 30, 37). Notably, the SCP was most affected in NMOSD patients, with the studies examining this plexus demonstrating a more prominent reduction in vessel density (13, 17, 21, 25, 27, 28, 31, 34, 36–39). Furthermore, ON + eyes exhibited a more pronounced reduction in vessel density compared to ON- eyes (13, 17, 21, 27, 28, 31, 35, 37, 38). Specifically, Chen et al. (31) observed that the SCP vessel density in ON + patients was around 37%, compared to 44% in those without previous optic neuritis attacks and 47% in healthy controls. In addition, Kwapong et al. (37) reported a strong correlation (*p* < 0.05) between the reduced capillary density in both the superficial and deep plexi and the number of optic neuritis attacks. In terms of the DCP, a controversy appears, with some studies showing that the vessel density is predominantly preserved in NMOSD patients, whereas others observed a reduction, and Lee et al. (36) reported a decrease only in the inferior quadrant. Additionally, Liu et al. (25) reported that DCP vessel density was reduced in NMOSD patients at baseline, but in the long term, it did not decline significantly, whereas SCP vessel density did decline longitudinally.

## Perfusion density

Some studies assessed macular perfusion density (PD) in addition to vessel density, in eyes with NMOSD, as the total area of perfused vessels per unit area in the parafoveal region (27, 38, 41, 42). Zhang et al. (38) reported a significant reduction of macular PD in NMOSD patients compared to healthy controls, while Dong et al. (42) also found a significantly lower optic-disc PD in NMOSD compared with healthy controls. In contrast, Liu et al. (41) did not report a significant difference in PD between groups but suggested that PD could have a value as part of a multimodal biomarker assessment of neuroaxonal injury and differentiation between MS and NMOSD. Furthermore, Zhang et al. (38) identified a strong correlation between vessel density and perfusion density, strongly highlighting its significance for accurate diagnosis.

## Foveal avascular zone

Foveal avascular zone (FAZ) findings are inconsistent across studies and should therefore be interpreted with caution. In some studies, an enlargement of the FAΖ was observed in NMOSD patients, regardless of optic neuritis status, compared to healthy controls (13, 21, 25, 27, 34). This difference persisted even when only NMOSD ON- patients were examined (13, 21, 34). The FAZ area reported by Lang et al. (13) was statistically significantly larger in both NMOSD ON+ (0.420mm^2^) and ON- patients (0.345 mm^2^) compared to healthy controls (0.273mm^2^). Ηowever, these findings were not confirmed by Zhang et al. (38), who found that NMOSD eyes had an approximately 0.05 mm^2^ smaller FAΖ area than healthy controls, a finding also supported by Tiftikcioglu et al. (40), who reported significantly reduced FAΖ size in both ON + and ON- NMOSD eyes. Dong et al. (42) did not report any statistically significant difference in FAZ area among groups (*p* = 0.083 and *p* = 0.789). The meta-analysis by Amanollahi et al. (43) found no consistent overall FAZ difference, supporting the view that FAZ should currently be considered an exploratory OCTA parameter rather than a reliable diagnostic or monitoring biomarker in NMOSD.

## Comparative OCTA findings in NMOSD, MOGAD, and MS

**Figure 2:**
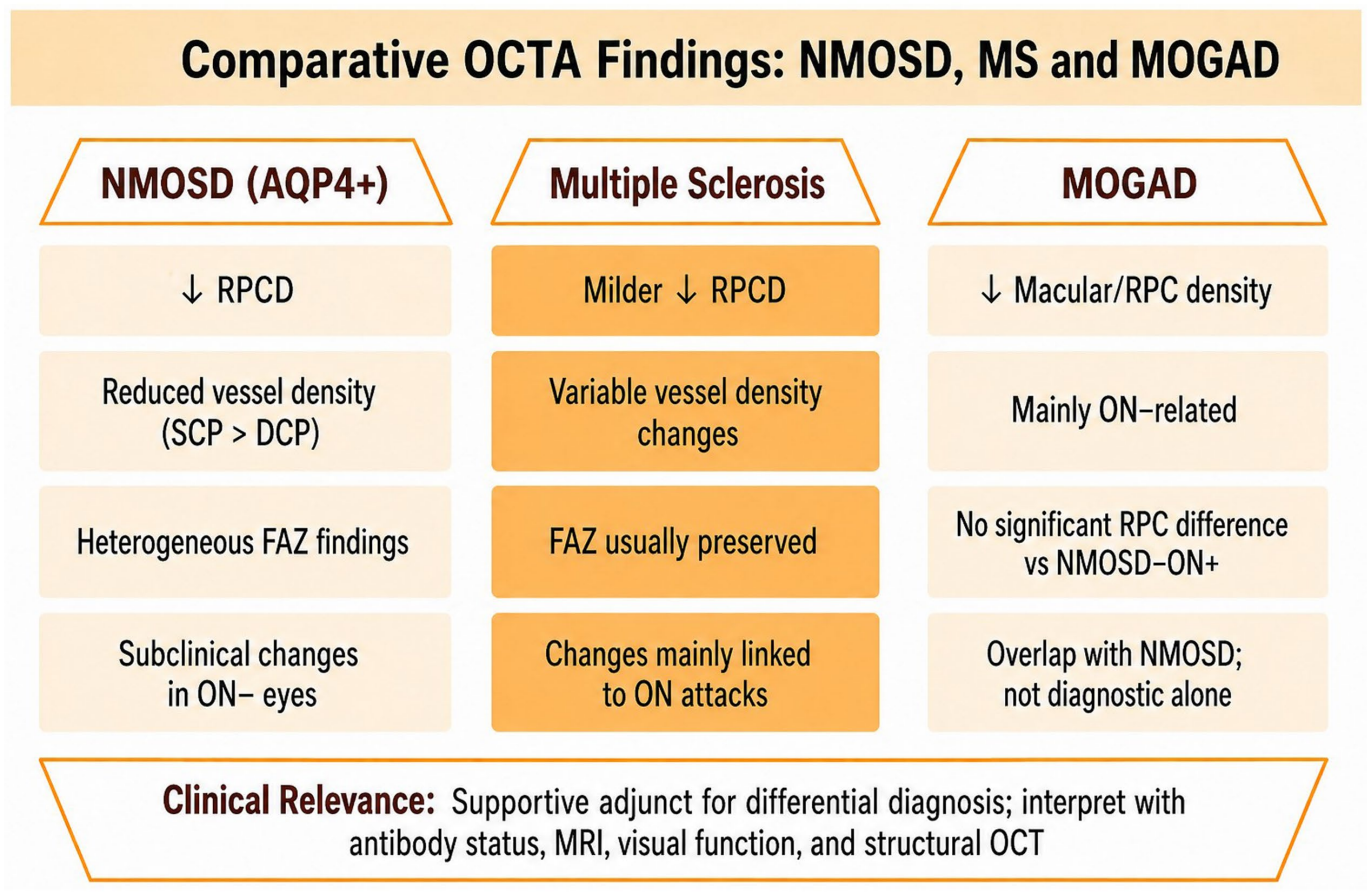
Comparative OCTA findings in NMOSD, MS, and MOGAD. OCTA demonstrates both potentially distinct patterns and important overlaps of retinal microvascular involvement across NMOSD, MS, and MOGAD, highlighting the clinical relevance of OCTA as a supportive tool within a multimodal diagnostic framework.

Comparative OCTA data are important because retinal microvascular abnormalities may occur across inflammatory demyelinating disorders. Lang et al. (13) directly compared OCTA findings in MOGAD and NMOSD and showed that both disorders may present retinal structural and microvascular abnormalities, although the patterns were not identical. Compared with NMOSD patients, MOGAD patients showed lower superficial vascular plexus density, whereas NMOSD patients showed larger FAZ measurements. When stratified by ON history, both MOGAD ON+ and NMOSD ON+ eyes showed reduced superficial vascular plexus density compared with healthy controls, indicating that ON-related damage contributes substantially to microvascular abnormalities in both conditions. The 2024 systematic review and meta-analysis by Amanollahi et al. (43) further supports this broader interpretation. This meta-analysis confirmed that SCP vessel density and RPCD were reduced in both NMOSD and MOGAD eyes. Importantly, in the ON+ subgroup comparison, Amanollahi et al. found no significant difference in RPC vessel density between NMOSD ON+ and MOGAD ON+ eyes. This suggests that peripapillary capillary rarefaction after optic neuritis may overlap between these two antibody-mediated disorders, limiting the disease specificity of this OCTA parameter. In MS, OCTA studies have also reported retinal microvascular alterations, generally interpreted in the context of demyelination, neuroaxonal loss, and ON. Comparative analyses have identified partly distinct macular and peripapillary vessel-density patterns between MS and NMOSD (26–28). These findings suggest that OCTA abnormalities may partly reflect shared effects of ON, retinal neuroaxonal loss, and inflammatory demyelination. Therefore, OCTA may contribute to multimodal differentiation among NMOSD, MS, and MOGAD, but only when interpreted together with antibody status, MRI, clinical phenotype, visual function, and structural OCT. These overlapping and partly distinct patterns are summarized schematically in Figure 2.

## Discussion

Neuromyelitis optica spectrum disorder is an autoimmune astrocytopathy of the CNS (2). The frequency of NMOSD is estimated to range from 0.3 to 4.4 cases per 100,000 individuals (18). Primary presentations include optic neuritis and myelitis; however, other manifestations, such as area postrema syndrome, have also been identified (3, 10, 11). The presence of AQP4-IgG has significantly transformed the diagnostic approach for patients (10). However, the diagnosis of seronegative NMOSD patients remains a challenge (2, 11).

The pathophysiology of NMOSD is primarily driven by autoimmune inflammation directed against AQP4 receptors (18). AQP4 is a transmembrane water channel mostly found in specific regions of the CNS, like the optic nerve and spinal cord, which are the principal target organs of the disease (11, 18). AQP4 is also primarily located on the foot processes of astrocytes adjacent to blood vessels, as well as in other cells with supportive role, such as the endfeet of Müller cells in the retina (18–20). Antibodies against AQP4 bind with the protein, activating complement-mediated cytotoxicity (18, 24). This leads to the breakdown of the blood–brain barrier, allowing infiltration by T cells, *Β* cells, and natural killer cells (18, 24). As a result of the excessive aggregation of lymphocytes, myelin-producing oligodendrocytes are damaged, leading to subsequent demyelination and neuronal death in the previously identified vulnerable regions (18, 24).

Antibody status is important when interpreting OCTA findings in NMOSD. Although the proposed mechanism of retinal microvascular injury is biologically linked to AQP4-IgG-mediated astrocytic and Müller cell dysfunction, the available OCTA literature does not consistently prove that the observed vascular alterations are specific to AQP4 + disease. Most included studies enrolled only AQP4 + patients or did not perform serostatus-stratified analyses. Dong et al. (42) addressed this limitation by comparing AQP4 + NMOSD with AQP4/MOG antibody seronegative NMOSD patients (double-seronegative). Differences between these groups were limited to selected optic-disc and macular OCTA parameters, while most OCTA measures did not differ significantly between subgroups. Therefore, the current evidence supports an association between NMOSD and retinal microvascular alteration but does not yet establish a consistent AQP4-specific OCTA phenotype. Future studies should separately analyze AQP4 + NMOSD, AQP4- NMOSD, MOGAD, and double-seronegative disease using standardized OCTA acquisition and segmentation protocols.

Moreover, OCTA does not directly measure astrocytic or Müller cell structure or function. OCTA-derived vessel density and perfusion metrics reflect retinal blood-flow signal and capillary architecture; therefore, any link between OCTA abnormalities and astrocytopathy remains inferential. The association between superficial vessel loss and serum GFAP reported by Aly et al. (21) supports a possible relationship between retinal microvascular alteration and astrocytic injury, but GFAP remains an indirect marker and does not establish causality.

**Figure 3:**
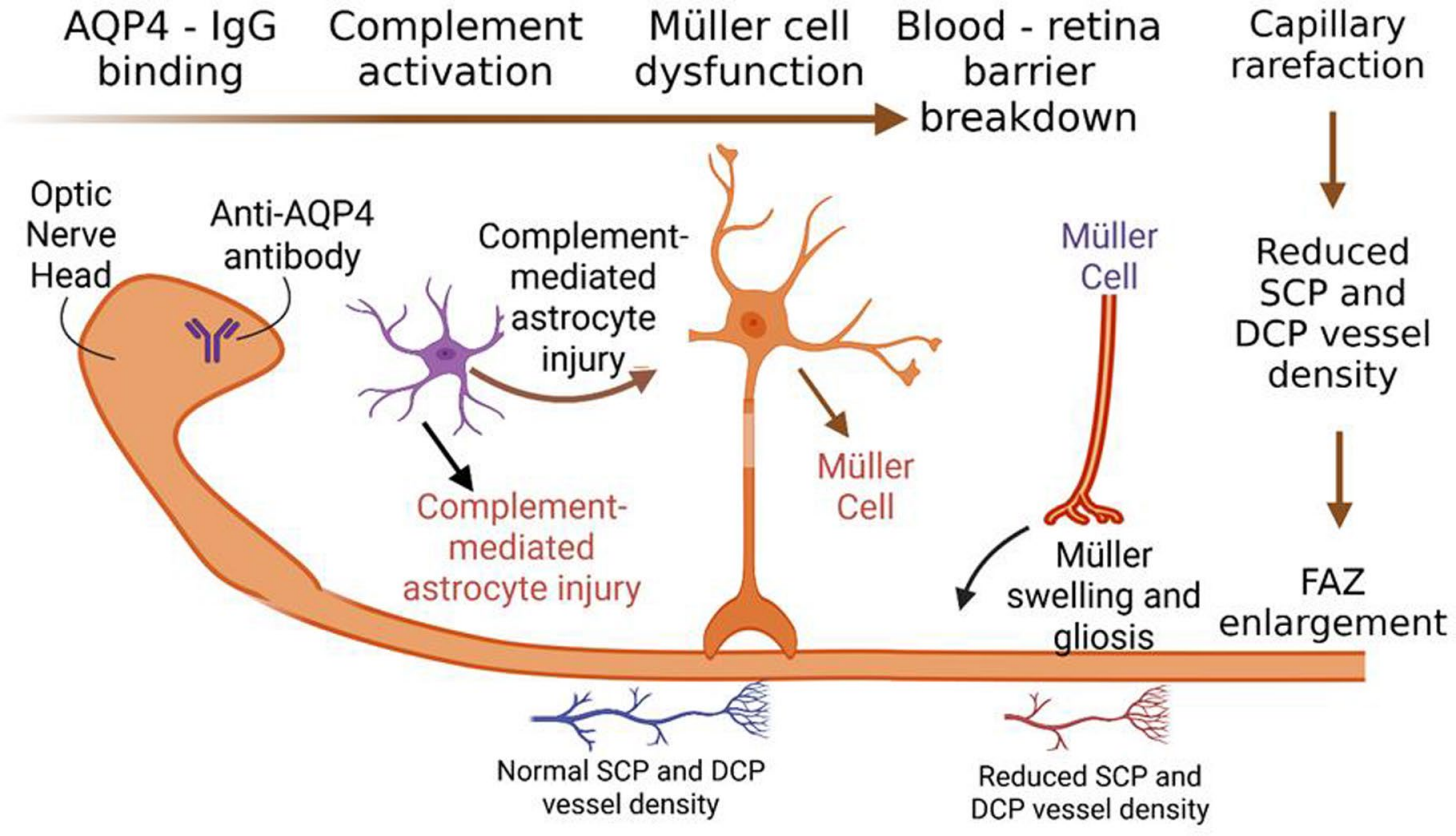
Proposed pathophysiological cascade of AQP4-IgG-mediated retinal involvement in neuromyelitis optica spectrum disorder. The schematic illustrates the sequence of cellular and vascular changes linking AQP4-IgG-mediated astrocyte injury to microvascular alterations detectable by optical coherence tomography angiography.

To be explicit, the retinal changes observed in NMOSD, such as the reduced vessel density and macular perfusion, may be partly explained by the secondary effects of this autoimmune attack as the optic nerve head is an extension of the CNS and shares similar vascular characteristics (15–18, 21, 24, 31). More precisely, the reduction in RPCD is likely a result of ischemic damage due to inflammation in the optic nerve and adjacent retinal layers (30, 31, 36, 44, 45). Moreover, optic nerve inflammation results in impaired axonal transport, leading to retinal ganglion cell death and thinning of the retinal nerve fiber layer (RNFL) (44, 45). The subsequent reduction in metabolic demand, together with possible trans-synaptic degeneration along the visual pathway, may contribute to secondary vascular rarefaction and decreased OCTA vessel density (30, 31, 36, 44, 45). Similarly, the reduction in macular vessel density, particularly in the superficial capillary plexus, can be attributed to ischaemic damage and inflammation affecting the retinal microcirculation (17, 18, 21, 24, 31, 37). The apparent predominance of superficial plexus involvement may be explained by its close anatomical relationship with the RNFL and ganglion cell layer, whereas the deep capillary plexus is located deeper and may be less directly linked to optic nerve axonal injury (17, 46). However, differences between superficial and deep plexus findings may also be influenced by OCTA segmentation boundaries, projection artifacts, image quality, and device-specific algorithms. FAΖ enlargement may reflect perifoveal capillary dropout secondary to astrocyte mediated vascular instability rather than a purely compensatory response (13, 21, 34). The proposed pathophysiological cascade linking AQP4-IgG-mediated astrocyte injury to Müller cell dysfunction and OCTA-detectable microvascular alterations is illustrated in Figure 3.

The main characteristics and key OCTA findings of the included studies are summarized in Table 1. Overall, the studies shared comparable inclusion and exclusion criteria based on the revised 2015 diagnostic criteria for NMOSD although differences in serostatus stratification and OCTA acquisition protocols were noted (11). Some of the studies classified NMOSD patients into two groups based on the presence of previous optic neuritis attacks and compared these groups with healthy controls of corresponding demographic characteristics (13, 17, 21, 26, 28, 30, 31, 34, 37). In contrast, other studies considered the serostatus for AQP4 when analyzing their results (21, 36). In general, the examined studies assessed similar factors using OCTA between the groups; however, they did not reach the exact same conclusions. According to the analysis, the main factors that were examined were the RPCD, the macular VD, the PD, and the size of the FAZ.

Specifically, in NMOSD patients, despite the history of optic neuritis, a decrease in the superficial vascular network density, peripapillary, or perifoveally, was reported when compared to healthy controls (13, 17, 26, 28, 30–32, 34–40). Among the groups with respect to optic neuritis history, a more pronounced reduction in perifoveal vessel density in favor of the ON + group was demonstrated (21, 30, 31, 36, 37). Kwapong et al. (37) and Lang et al. (13) also reported a correlation with the number of previous optic neuritis episodes (*p* < 0.05). However, a reduction of vessel density was also observed in ON- patients when compared to healthy controls (17, 21, 30, 31, 34). Wei et al. (17), for instance, demonstrated a lower DCP density in the NMOSD ON- group (*p* = 0.007), an observation that was also evident in the SCP, albeit to a lesser extent, when compared to healthy controls. An impact on the DCP density in NMOSD was suggested from more than one study (13, 34, 36, 37).

This reported reduction in RPCD and macular VD may be explained through the disease’s pathophysiological mechanisms. Two hypotheses have been put forward to account for this phenomenon in the studies cited above. The first suggests that the decrease in vessel density within the SCP could be linked to optic neuritis itself, where the shrinkage of the capillary network occurs as a result of nerve fiber and ganglion cell atrophy, due to the decrease in metabolic demands (44, 45). Consequently, ON + patients tend to have lower vessel density, which may be further exacerbated by recurrent episodes of optic neuritis. *A* hypothesis that appears to be supported by the analyzed studies, as patients with NMOSD exhibited lower vessel density following previous episodes of optic neuritis.

Ηowever, the observed reduction in vessel density in ON-patients, along with the involvement of the deep capillary plexus in some cases, raises the question whether vessel loss could be a primary feature of NMOSD itself, independent of optic neuritis. There is no clear answer, but the presence of AQP4-IgG may explain this observation (18, 24). So, regarding the pathophysiology of the disease, evidence suggests that the reduction in vessel density may be due to direct vascular damage or secondary to the overall inflammatory environment induced by AQP4-IgG (18, 21, 24). Then, inflammation causes further ischemic injury and ultimately damage to neural tissue, even in areas outside of the optic nerves (18, 24). Aly et al. (21) reported that microvascular retinal changes in NMOSD patients, independent of a history of ON, were significantly associated with elevated plasma levels of glial fibrillary acidic protein (GFAP), a biomarker indicative of astrocytic injury. These findings align with the underlying pathophysiology of NMOSD, which is characterized by astrocytopathy and support the concept that retinal microvascular alterations in NMOSD are not exclusively a consequence of optic neuritis but may partly reflect a AQP4-IgG–related astrocytic injury (18, 24). However, because OCTA does not directly image glial pathology and serostatus-stratified OCTA data remain limited, this mechanism should be interpreted as plausible rather than proven.

The FAZ size, meaning the area of the retina that lacks blood vessels, displayed controversy between the examined studies, with some of them showing an enlargement while others a restriction or no difference between the studied groups (13, 21, 25, 27, 34, 38, 40, 42). Changes in its size or shape can indicate retinal vascular abnormalities (16, 46). The enlargement of FAΖ size in NMOSD patients may be explained by the pathophysiological mechanisms of the disease. This may occur due to the loss of vasculature caused by recurrent episodes of optic neuritis or due to the underlying autoimmune processes of the disease triggered by AQP4 autoantibodies, even in the absence of clinically apparent ON (13, 18, 21, 24, 34). Specifically, for the latter, it has been hypothesized that AQP4 channels in Müller cells are targeted by *A*QP4-IgG, leading to disruption of the blood–retinal barrier as a result of Müller cell loss and decreased retinal vascular density, thereby contributing to the enlargement of the FAΖ (18–21). However, some studies have found that FAΖ size in NMOSD patients was smaller compared to healthy controls, though this finding is less common (38, 40). A smaller FAΖ could be interpreted as a compensatory vascular change in response to ischemia and reduced blood supply to the retina (38). This suggests that a reduction in blood supply leads to the remodeling of capillaries adjacent to the avascular zone, resulting in elongation of the vessels and, consequently, a smaller FAΖ (38).

The autoimmune attack may lead to complex changes in the retinal blood flow and perifoveal microvascular remodeling, which could contribute to FAΖ alterations (18, 24). Therefore, it could be hypothesized that FAZ variability may partly reflect differences in disease stage, with FAZ enlargement potentially occurring during acute or more severe microvascular injury and smaller FAZ measurements, possibly reflecting remission-related vascular remodeling. However, this interpretation remains speculative. The studies that have observed a smaller FAΖ generally involved a larger sample size compared to those with opposite findings; however, the stage of the disease was not specified in these studies (38, 40). It is essential to note that the observation of a smaller FAΖ is not frequently reported, and there is no consensus on whether it is a consistent characteristic of NMOSD. This lack of consistency is also supported by the meta-analysis by Amanollahi et al., which did not confirm a consistent overall FAZ difference. Therefore, FAZ should currently be considered an exploratory OCTA parameter, and further research is needed to better understand its significance in the pathophysiology of the disease.

Several methodological and biological factors may explain the conflicting FAZ results. FAZ measurements are highly sensitive to OCTA scan size, device software, segmentation boundaries, image quality, FAZ delineation algorithms, sample size, and statistical adjustment. Differences in disease duration, disease stage, time from last ON attack, number of previous ON attacks, treatment status, antibody status, inclusion of seronegative or MOGAD cases, and inclusion of ON+ versus ON− eyes may also influence FAZ morphology. These sources of heterogeneity limit the comparability of FAZ findings and prevent FAZ from being considered a validated NMOSD biomarker of disease severity, relapse frequency, progression, or treatment response at present.

Finally, perfusion density was the least measured parameter, making it difficult to draw definitive conclusions regarding its clinical significance. Although the analyzed studies suggest that PD may have value as part of a multimodal biomarker approach for assessing NMOSD, further longitudinal investigation is needed before its role can be established (38, 41, 42).

The differential diagnostic value of OCTA should be considered in the broader context of inflammatory demyelinating disorders, particularly MS and MOGAD. Based on the aforementioned studies, vessel density biomarkers on OCTA that appear reduced in the peripapillary or macular region even in patients without a history of optic neuritis may serve as a valuable clinical marker to support the diagnosis of NMOSD and its differentiation from MS. The different OCTA patterns seen in NMOSD compared with multiple sclerosis are illustrated schematically in Figure 2.

Regarding MOGAD, the available comparative evidence suggests that OCTA abnormalities may overlap with those observed in NMOSD, particularly in eyes with previous optic neuritis (13, 43, 47). Although some differences in vascular patterns have been reported between these disorders, reduced vessel density in both conditions may partly reflect shared consequences of optic neuritis, retinal neuroaxonal loss, and inflammatory demyelination (13, 43, 47). OCTA may therefore serve as a supportive component of multimodal differential assessment in NMOSD, MS, and MOGAD, provided that its findings are interpreted alongside antibody status, MRI features, clinical phenotype, visual function, and structural OCT.

Several studies have also reported that lower retinal vessel density on OCTA is associated with worse visual acuity and higher scores on the expanded disability status scale (EDSS) (17, 21, 29, 31, 36, 37). As these microvascular alterations have been detected even in ON− eyes, OCTA may help identify subclinical retinal involvement and establish a retinal microvascular baseline for longitudinal follow-up, although its role in tracking disease progression remains unvalidated (17, 21, 30, 31, 34). In addition, Ma et al. (32) showed that microvascular alterations in the peripapillary region were linked to functional connectivity in the primary visual cortex, indicating that optic neuritis-related damage identified with OCTA may reflect degenerative changes within the visual cortex.

Although most studies report reduced vessel density in patients with NMOSD, some inconsistencies appear, particularly in results related to macular perfusion and the size of the FAZ (13, 17, 21, 26, 28, 30–32, 34–40). These differences may be explained by variations in study design, patient populations, and imaging procedures (22, 46, 48). For instance, certain studies examined only patients who had experienced ON, while others also included individuals without a history of ON (21, 30, 31, 36, 37). As vascular alterations linked to ON may be more marked, this difference in patient selection could influence the findings.

Differences in OCTA technology may also have played a role. Various OCTA devices offer different levels of image resolution, which can affect how accurately parameters such as VD and FAZ size are measured (22, 46). In addition, some studies applied distinct imaging protocols or analytical approaches when processing OCTA data, which may further contribute to the variation in results. Establishing standardized OCTA protocols—covering both device characteristics and data analysis methods—would help improve the consistency and comparability of findings across studies.

Another element that may affect the results is the stage of the disease when imaging takes place. Since NMOSD follows a relapsing course, vascular measurements may vary depending on disease activity at the time of assessment (1, 4). Retinal vascular changes, for example, may vary and potentially be more evident during or soon after an acute relapse than during a remission, although this has not been adequately evaluated. Long-term studies following patients over time are therefore needed to clarify how these vascular alterations develop and how they relate to disease activity and treatment.

OCTA holds significant promise as a non-invasive imaging tool for advancing the understanding and management of NMOSD (16, 46). Addressing the key limitations of existing literature is essential to determine whether OCTA-derived metrics can serve as reliable diagnostic, prognostic, or monitoring biomarkers for NMOSD (22). These retinal abnormalities seen on OCTA may allow early detection of subclinical retinal involvement in NMOSD even in the absence of optic neuritis and may provide a retinal marker of broader CNS involvement (17, 21, 32, 33). Thus, future research should focus on clarifying OCTA’s role in early detection of NMOSD and understanding the retinal microcirculation in NMOSD. Measuring these vascular alterations may allow OCTA to be explored as a potential prognostic indicator in NMOSD, helping to assess disease stage, severity, progression, and relapse patterns. Such measurements may also relate to visual acuity and overall morbidity, offering useful insight into how the disease may affect patients’ quality of life. In addition, OCTA-derived vessel density measures may be explored as imaging endpoints in future clinical trials, provided that standardized acquisition protocols, longitudinal follow-up, and treatment-stratified analyses are used. However, OCTA is not yet validated for relapse prediction, prognostication, treatment-response monitoring, or routine trial-endpoint use in clinical practice.

Further investigation into the mechanisms behind retinal vascular injury—especially those linked to AQP4 autoimmunity—could improve understanding of the broader systemic effects of NMOSD. To strengthen the clinical value of OCTA, future research should involve larger and more diverse patient cohorts, include multicentre data, use standardized OCTA protocols, and carefully control for ocular and systemic confounders. These steps may support more accurate diagnosis, improved understanding of prognosis and disease progression, and a deeper understanding of the disease’s underlying mechanisms. Overall, these directions highlight the importance of a comprehensive approach to the study and management of NMOSD.

## Potential clinical role of OCTA in NMOSD

OCTA should not be considered a replacement for clinical assessment, MRI, AQP4-IgG and MOG-IgG testing, visual acuity, visual field testing, or structural OCT measurements, such as RNFL and GCL thickness. Instead, OCTA may provide complementary information by assessing retinal microvasculature in a layer-specific manner. Structural OCT may identify neuroaxonal loss, whereas OCTA may help characterize whether vascular rarefaction precedes, accompanies, or follows this damage.

A practical clinical framework would be as follows. Suspected NMOSD should be evaluated with clinical history, neurological examination, MRI of the brain, spinal cord and optic nerves, AQP4-IgG testing, and MOG-IgG testing. Ophthalmic assessment should be performed, such as visual acuity, visual field testing, OCT-derived RNFL and ganglion cell layer (GCL) measurements, and OCTA when image quality is sufficient. OCTA should ideally be performed alongside structural OCT, preferably outside the acute phase of ON, to establish both structural and microvascular retinal baselines. In ON+ eyes, reduced vessel density should be interpreted in relation to post-inflammatory neuroaxonal loss. In ON− eyes, reduced vessel density may suggest subclinical retinal involvement, but it should not be considered diagnostic on its own. During follow-up, OCTA should be repeated using the same device, scan size, and segmentation protocol, and interpreted together with relapse history, EDSS, visual function, OCT structural measures, MRI activity, antibody status, treatment exposure, and clinical course. FAZ measurements should remain exploratory and should not currently guide clinical decision-making.

Recent longitudinal data showing progressive RPC and SCP density reduction over time in AQP4 + NMOSD, as well as associations between RPC density and white matter hyperintensity burden, support the potential role of OCTA as a candidate longitudinal monitoring biomarker. However, these evidence remains observational and does not establish causality or validate OCTA as a treatment-response tool (25).

## Limitations and future directions

As identified across the reviewed studies, OCTA has shown promising results in NMOSD, but with several limitations. First, the technique’s sensitivity to motion artifacts and segmentation errors can compromise image quality and accuracy (22, 46). This may be especially relevant in NMOSD patients with poor visual acuity, in whom fixation instability can reduce scan reliability (46). Second, there is notable heterogeneity in OCTA protocols, analysis software, and definitions of retinal layers across studies, which limits comparability and standardization (22, 48). Future work should focus on standardizng segmentation protocols and reporting metrics across devices. Development of automated analysis tools is also critical to facilitate the integration of OCTA biomarkers into clinical decision-making. Many studies also lacked longitudinal follow-up, which makes it difficult to determine whether OCTA can reliably predict disease progression or treatment response. This limitation highlights the need for long-term investigations and multicentre studies to better evaluate the predictive role of OCTA in NMOSD.

Beyond these technical and longitudinal limitations, OCTA-derived VD, PD, and FAZ measurements may also be influenced by ocular and systemic confounders. Glaucoma, diabetic or hypertensive retinopathy, retinal vascular disease, high myopia or axial length differences, media opacity, cardiovascular disease, and treatment exposure can all affect retinal microvascular measurements. Several included studies attempted to reduce these effects by excluding ocular disease, poor OCTA image quality, recent optic neuritis, or significant refractive error. For example, Dong et al. (42) excluded MOGAD+ cases, ON recurrence within the previous 6 months, glaucoma, macular degeneration, severe lenticular opacity, marked refractive error, inability to cooperate with OCT examination, and low OCTA scan quality. Liu et al. (25) also excluded severe glaucoma, age-related macular degeneration, poor OCTA image quality, and other confounding ocular or neurological disorders. Nevertheless, confounder control was not uniform across studies, and residual confounding remains possible. Therefore, retinal microvascular rarefaction cannot be attributed exclusively to NMOSD-related astrocytopathy unless ocular, systemic, and structural neuroaxonal confounders are carefully excluded or adjusted for.

It is also important to note that OCTA mainly reflects vascular and structural alterations within the retina and may not fully represent the extent of central nervous system involvement in NMOSD, particularly in patients with brain or spinal cord lesions (11, 24, 32). Combining OCTA measurements with other markers—such as MRI findings or serum GFAP levels—could help clarify the relationship between retinal changes and CNS pathology and provide a more comprehensive view of the disease (21, 32, 39). In addition, reduced vessel density may reflect primary microvascular involvement, but it may also represent reduced metabolic demand secondary to retinal ganglion cell or axonal loss after optic neuritis; therefore, OCTA findings should be interpreted together with RNFL/GCL thickness and visual function.

Another limitation is that only a small number of studies directly compare NMOSD with MOGAD, and available meta-analytic evidence suggests overlapping OCTA abnormalities between NMOSD and MOGAD, particularly after ON. Therefore, disease-specific OCTA signatures remain insufficiently defined.

Finally, the effects of disease stage, treatment status, and previous relapses on OCTA parameters remain insufficiently understood. Further prospective, well-controlled studies are therefore needed. Standardizing OCTA biomarkers and validating them in large-scale cohorts will be important steps toward improving their clinical usefulness (23).

## Conclusion

OCTA provides detailed information about the retinal microvasculature. Current evidence supports OCTA as a promising adjunctive imaging modality in NMOSD, particularly for evaluating retinal vessel density in relation to optic neuritis history, structural OCT damage, visual function, and disability. Reduced vessel density, especially in the superficial macular and radial peripapillary capillary networks, appears to be the most consistent OCTA finding and may provide complementary information about ocular involvement and broader disease burden. OCTA may help establish a retinal microvascular baseline, identify possible subclinical retinal involvement in ON− eyes, support differentiation from MS and MOGAD in selected contexts, and contribute to longitudinal follow-up when interpreted as part of an integrated neurologic and ophthalmic assessment. However, OCTA is not yet validated as a stand-alone diagnostic, monitoring, or treatment response tool. Its clinical use should remain complementary to established investigations. FAZ findings remain heterogeneous and should currently be considered exploratory. In addition, serostatus-specific evidence remains limited, and OCTA changes must be interpreted cautiously because ocular/systemic confounders and optic neuritis-related neuroaxonal loss may influence vascular measurements. Larger longitudinal studies using standardized OCTA protocols, detailed antibody stratification, and rigorous confounder control are required before OCTA-derived biomarkers can be incorporated into routine NMOSD decision-making.
